# Nomenclature report on rice WRKY's - Conflict regarding gene names and its solution

**DOI:** 10.1186/1939-8433-5-3

**Published:** 2012-02-27

**Keywords:** Gene nomenclature, WRKY, family gene, rice

## Abstract

**Background:**

Since whole genome sequences of rice were made publically accessible, the number of articles on new rice genes has increased remarkably. The Committee on Gene Symbolization, Nomenclature and Linkage (CGSNL) of the Rice Genetics Cooperative published the gene nomenclature system for rice and encouraged researchers to follow the rules before publishing their results. The CGSNL provides an on-line registration system for newly identified rice genes to prevent conflicts and/or duplication of gene name in journal articles.

**Findings:**

Recently, the CGSNL surveyed genes in the rice WRKY family in published journal articles and found several duplicated gene names.

**Conclusions:**

To discuss and resolve inconsistencies in WRKY gene nomenclature, the rice WRKY working group was established and redefined the nomenclature. This report announces the conclusion.

## 

Since 2004, when whole genome sequences of rice were made publically accessible (Goff et al. 2002; Yu et al. 2002; IRGSP 2005; Yuan et al. 2003, 2005), the number of articles on new rice genes, including genome-wide studies of rice gene families, has increased remarkably. Although vigorous research activity is promising, the conflicts with regard to the duplication of gene names in journal articles can occur. Therefore, the CGSNL (Committee on Gene Symbolization, Nomenclature and Linkage, Rice Genetics Cooperative) published the "Gene Nomenclature System for Rice" in 2008 (CGSNL 2008) and encouraged researchers to follow the nomenclature rules before publication of their results.

The WRKY family is one of the largest families of transcription factors in higher plants and WRKY genes have key roles in plant development and responses to environmental stresses (see Rushton et al. 2010 and Chen et al. 2011 for recent reviews). Recently, the CGSNL surveyed genes in the rice WRKY family in published journal articles and found several duplicated gene names (Additional file 1: Table S1). Several reasons account for such duplications. For example, if two research groups independently performed their studies and submitted the results to different journals at the same time, neither group would be aware of the results of the other research group until the studies were published. In some cases, a sequence in the DNA data banks and/or in the genome databases was updated after the publication of a study, and only the older version of the sequence contained the WRKY domain, indicating that the gene became obsolete (retired from new gene list, e.g. WRKY78 in Additional file 1: Table S1). Hence, researchers would not be able to find the original sequence of that gene in the database. Alternatively, a researcher might name a new gene by placing a prefix "Os (Oryza sativa)" followed by the name of the orthologous Arabidopsis gene, and another researcher might name the same new gene according to the rice nomenclature rule; this would cause the new gene to have two or more different names. Moreover, if an obsolete gene name was reused for a new gene by other researchers, it might lead to confusion. In a remarkably rapidly advancing field of research, although such conflicts are apt to happen, it may mislead researchers who attempt to utilize published but misused gene names.

To resolve the conflicts and confusion in WRKY gene names and symbols, the CGSNL established a rice WRKY-working group that includes corresponding authors (Qiu et al. 2004; Zhang et al. 2004; Wu et al 2005; Xie et al. 2005; Zhang and Wang 2005; Ryu et al. 2006; Ross et al. 2007; Berri et al. 2009), and redefined the gene names as CGSNL proposal main genes, as shown in Table S1. First, based on the CGSNL rules, the gene names were arranged in numerical order according to the date of publication and assigned gene names on the basis of the numerical order. To document the publication records, we started from Zhang et al. (2004) as this is the first publication on rice WRKY family genes (numbered from 1 to 77). The second paper, Qui et al. (2004), published 97 WRKY genes. Based on our investigation, WRKY38, -44, -59, -63, and WRKY78 to -97 were novel genes. Hence, new numbers were assigned for WRKY38, -44, -59, and -63 because these numbers have been already used. In such a way, we have examined publications in chronological order and assigned new numbers when necessary. Columns B and C show the locus numbers recently provided by RAP and MSU (TIGR), respectively. Blanks in these columns mean obsolete genes, unannotated genes, or genes present only in the *indica *subspecies. We stress here that the objective of this proposal is to resolve the nomenclature of published gene names, but not to review if a specified gene is a bona fide WRKY gene or not. Also, it is very likely that the genome sequence will be updated going forward. Therefore, the naming system proposed here needs to be revised in the future.

Table S1 lists the gene names as per this convention for the rice WRKY gene family members. We request authors utilize this gene nomenclature and bring to our attention any concerns if a gene name has not been allocated according to its first reported instance. We hope that all researchers will submit new gene names through the online submission system (http://www.shigen.nig.ac.jp/rice/oryzabase_submission/gene_nomenclature/) prior to publishing their results and add a sentence in future publications. For example, "WRKY genes in the present study are named according to the CGSNL nomenclature" as part of either the footnote, acknowledgements and/or methods section as deemed fit by the journal publishers. We hope that this practice will become generally acceptable to the scientific community. We also request that journal editors discourage authors from publishing duplicate names for the same genes by encouraging authors to register and/or confirm their gene names and symbols by contacting the CGSNL via the online portal. Your cooperation will greatly contribute to a productive research progress and consistency in reporting.

## List of abbreviations

RAP: Rice Annotation Project; TIGR: The Institute for Genomic Research; MSU: Michigan State University.

## Competing interests

The authors declare that they have no competing interests.

## Authors' contributions

QJS,DY,JJ,PP,PA,ZG and YZ, authors of rice WRKY gene original papers, participated in re-naming their original gene names. TI,SSL and CRB, members of databases, participated in assigning locus ID to each genes. YN,SM,MY,GW,KKJ,LX,BM,PJ and YY, members of CGSNL, participated in establishment of this working group and discussion on the solution of gene name conflict. YY drafted the manuscript and BM, CRB, PJ, DY, PA contributed to revise and improve it. All authors read and approved the final manuscript.

## Supplementary Material

Additional file 1 **Table S1. The WRKY family genes in *Oryza sativa *L. ssp. *japonica***. Columns B and C list genomic locus ID of RAP and MSU (TIGR), respectively. Columns D and E show the proposal name of main gene symbol and its synonym, respectively. Columns F to O, publications are listed in chronological order, show the gene names used in each publication. Yellow highlights indicate that the original name is the same as that proposed by the CGSNL. From row 98 to 125, names marked with a color that is same as that in row 2 indicates that the genes were renamed by the CGSNL due to duplication. For example, WRKY38 in column G was renamed as WRKY98. Rushton et al. (2010) is a recent review, not an original research publication. Data in columns N and O are shown for reference. The corresponding author of this review paper is the same as that in three original research papers (shown in columns F, H and L). Click here for file
