# Supplementary material for: Nomenclature report on rice WRKY's - Conflict regarding gene names and its solution
Source: Rice (N Y). 2012 Feb 27;5(1):3. doi: 10.1186/1939-8433-5-3 (PMC3834489; doi:10.1186/1939-8433-5-3)
Supplement: Supplementary file 1 [file 1939-8433-5-3-S1.DOC]

A	B	C	D	E	F	G	H	I	J	K	L	M	N	O
No.	RAP_ID	MSU(TIGR)_ID	**CGSNL proposal main gene symbol**	**CGSNL proposal synonym gene symbol**	Zhang et al. 2004 PMID: 15047897	Qiu et al. 2004 **DOI: 10.1360/ 982004-183**	Xie et al. 2005 PMID: 15618416	Zhang & Wang 2005 PMID: 15629062	Wu et al. 2005 PMID= 16106749	Ryu et al. 2006 PMID: 16528562	Ross et al. 2007 **DOI:10.1111/ j.1744-7909.2007.00504.x**	Berri et al. 2009 PMID: 19772648	Rushton 2010 review(tableS1)	Rushton 2010 review(tableS2)
1	Os01g0246700	LOC_Os01g14440	**WRKY 1**		WRKY1	WRKY1	WRKY1	WRKY96	4	WRKY1	WRKY1	WRKY96	WRKY1/ WRKY96	WRKY1
2	Os10g0579400	LOC_Os10g42850	**WRKY 2**		WRKY2	WRKY2	WRKY2	WRKY1	86	WRKY2	WRKY2	WRKY1	WRKY2/ WRKY1	WRKY2
3	Os03g0758000	LOC_Os03g55080	**WRKY 3**		WRKY3	WRKY3	WRKY3	WRKY3	37	WRKY3	WRKY3	WRKY3	WRKY3	WRKY3
4	Os03g0758900	LOC_Os03g55164	**WRKY 4**		WRKY4	WRKY4	WRKY4	WRKY2	36	WRKY4	WRKY4	WRKY2		WRKY4
5	Os05g0137500	LOC_Os05g04640	**WRKY 5**		WRKY5	WRKY5	WRKY5	WRKY43	46	WRKY5	WRKY5	WRKY43	WRKY5/ WRKY43	WRKY5
6	Os03g0798500	LOC_Os03g58420	**WRKY 6**		WRKY6	WRKY6	WRKY6	WRKY102	38	WRKY6	WRKY6	WRKY102		WRKY6
7	Os05g0537100	LOC_Os05g46020	**WRKY 7**		WRKY7	WRKY7	WRKY7	WRKY46	57	WRKY7	WRKY7	WRKY46	WRKY7	WRKY7
8	Os05g0583000	LOC_Os05g50610	**WRKY 8**		WRKY8	WRKY8	WRKY8	WRKY44	61	WRKY8	WRKY8	WRKY44	WRKY8/ WRKY44	WRKY8
9	Os01g0289666	LOC_Os01g18584	**WRKY 9**		WRKY9	WRKY9	WRKY9	WRKY88	6	WRKY9	WRKY9	WRKY88		WRKY9
10	Os01g0186000	LOC_Os01g09100	**WRKY 10**		WRKY10	WRKY10	WRKY10	WRKY27	3	WRKY10	WRKY10	WRKY27	WRKY10/WRKY27	WRKY10
11	Os01g0626400	LOC_Os01g43650	**WRKY 11**		WRKY11	WRKY11	WRKY11	WRKY24	10	WRKY11	WRKY11	WRKY24	WRKY11/WRKY24	WRKY11
12	Os01g0624700	LOC_Os01g43550	**WRKY 12**		WRKY12	WRKY12	WRKY12	WRKY23	9	WRKY12	WRKY12	WRKY23	WRKY12/WRKY23	WRKY12
13	Os01g0750100	LOC_Os01g54600	**WRKY 13**		WRKY13	WRKY13	WRKY13	WRKY84	16	WRKY13	WRKY13	WRKY84	WRKY13	WRKY13
14	Os01g0730700	LOC_Os01g53040	**WRKY 14**		WRKY14	WRKY14	WRKY14	WRKY33	14	WRKY14	WRKY14	WRKY33	WRKY14/WRKY33	WRKY14
15	Os01g0656400	LOC_Os01g46800	**WRKY 15**		WRKY15	WRKY15	WRKY15	WRKY97	11	WRKY15	WRKY15	WRKY97	WRKY15	WRKY15
16		LOC_Os01g47560	**WRKY 16**		WRKY16	WRKY16	WRKY16	WRKY35	12	WRKY16	WRKY16	WRKY35		WRKY16
17	Os01g0972800	LOC_Os01g74140	**WRKY 17**		WRKY17	WRKY17	WRKY17	WRKY28	24	WRKY17	WRKY17	WRKY28	WRKY17	WRKY17
18	Os10g0328400	LOC_Os10g18099	**WRKY 18**		WRKY18	WRKY18	WRKY18	WRKY65		WRKY18		WRKY65		WRKY99
19	Os05g0571200	LOC_Os05g49620	**WRKY 19**		WRKY19	WRKY19	WRKY19	WRKY107	60	WRKY19	WRKY19	WRKY110	WRKY19/WRKY11	WRKY19
20	Os01g0820900	LOC_Os01g60540	**WRKY 20**		WRKY20	WRKY20	WRKY20	WRKY40	19	WRKY20	WRKY20	WRKY40	WRKY20/WRKY40	WRKY20
21	Os01g0821600	LOC_Os01g60640	**WRKY 21**		WRKY21	WRKY21	WRKY21	WRKY68	21	WRKY21	WRKY21	WRKY68	WRKY21	WRKY21
22	Os01g0820400	LOC_Os01g60490	**WRKY 22**		WRKY22	WRKY22	WRKY22	WRKY38	17	WRKY22	WRKY22	WRKY38	WRKY22/WRKY38	WRKY22
23	Os01g0734000	LOC_Os01g53260	**WRKY 23**		WRKY23	WRKY23	WRKY23	WRKY31	15	WRKY23	WRKY23	WRKY31	WRKY23/WRKY31	WRKY23
24	Os01g0826400	LOC_Os01g61080	**WRKY 24**		WRKY24	WRKY24	WRKY24	WRKY22	22	WRKY24	WRKY24	WRKY22	WRKY24	WRKY24
25	Os08g0235800	LOC_Os08g13840	**WRKY 25**	**WRKY 44**	WRKY44		WRKY44		73	WRKY44				
26	Os08g0235800	LOC_Os08g13840	**WRKY 25**	**WRKY 44**	WRKY25	WRKY25	WRKY25	WRKY56	73	WRKY25	WRKY25	WRKY56		WRKY25
27	Os01g0714800	LOC_Os01g51690	**WRKY 26**	**WRKY 59**	WRKY26	WRKY26	WRKY26	WRKY36	13	WRKY26	WRKY26	WRKY36	WRKY26	WRKY26
28	Os01g0714800	LOC_Os01g51690	**WRKY 26**	**WRKY 59**	WRKY59		WRKY59		13	WRKY59				
29	Os01g0586800	LOC_Os01g40430	**WRKY 27**		WRKY27	WRKY27	WRKY27	WRKY32	8	WRKY27	WRKY27	WRKY32		WRKY27
30	Os06g0649000	LOC_Os06g44010	**WRKY 28**		WRKY28	WRKY28	WRKY28	WRKY4	66	WRKY28	WRKY28	WRKY4	WRKY28/WRKY4	WRKY28
31	Os07g0111400	LOC_Os07g02060	**WRKY 29**		WRKY29	WRKY29	WRKY29	WRKY49	67	WRKY29	WRKY29	WRKY49	WRKY29	WRKY29
32	Os08g0499300	LOC_Os08g38990	**WRKY 30**		WRKY30	WRKY30	WRKY30	WRKY47	76	WRKY30	WRKY30	WRKY47		WRKY30

A	B	C	D	E	F	G	H	I	J	K	L	M	N	O
No.	RAP_ID	MSU(TIGR)_ID	**CGSNL proposal main gene symbol**	**CGSNL proposal synonym gene symbol**	Zhang et al. 2004 PMID: 15047897	Qiu et al. 2004 **DOI: 10.1360/982004-183**	Xie et al. 2005 PMID: 15618416	Zhang & Wang 2005 PMID: 15629062	Wu et al. 2005 PMID=16106749	Ryu et al. 2006 PMID: 16528562	Ross et al. 2007 **DOI:10.1111/j.1744-7909.2007.00504.x**	Berri et al. 2009 PMID: 19772648	Rushton 2010 review(tableS1)	Rushton 2010 review(tableS2)
33	Os06g0504900	LOC_Os06g30860	**WRKY31**		WRKY31	WRKY31	WRKY31	WRKY6	65	WRKY31	WRKY31	WRKY6	WRKY31	WRKY31
34	Os02g0770500	LOC_Os02g53100	**WRKY32**		WRKY32	WRKY32	WRKY32	WRKY17	30	WRKY32	WRKY32	WRKY17		WRKY32
35			**WRKY33**		WRKY33	WRKY33	WRKY33			WRKY33				
36	Os02g0652100	LOC_Os02g43560	**WRKY34**		WRKY34	WRKY34	WRKY34	WRKY12	28	WRKY34	WRKY34	WRKY12	WRKY34	WRKY34
37	Os04g0471700	LOC_Os04g39570	**WRKY35**		WRKY35	WRKY35	WRKY35	WRKY37	41	WRKY35	WRKY35	WRKY37	WRKY35/WRKY37	WRKY35
38	Os04g0545000	LOC_Os04g46060	**WRKY36**		WRKY36	WRKY36	WRKY36		42	WRKY36	WRKY36	WRKY111		WRKY36
39	Os04g0597300	LOC_Os04g50920	**WRKY37**		WRKY37	WRKY37	WRKY37	WRKY34	43	WRKY37	WRKY37	WRKY34		
40			**WRKY38**		WRKY38		WRKY38			WRKY38				
41	Os02g0265200	LOC_Os02g16540	**WRKY39**		WRKY39	WRKY39	WRKY39	WRKY54	26	WRKY39	WRKY39	WRKY54	WRKY39	WRKY39
42	Os11g0117500	LOC_Os11g02530	**WRKY40**		WRKY40	WRKY40	WRKY40	WRKY10	90	WRKY40	WRKY40	WRKY10	WRKY40	WRKY40
43	Os11g0686400	LOC_Os11g45924	**WRKY41**		WRKY41	WRKY41	WRKY41			WRKY41	WRKY41			lacks WRKY signature
44	Os02g0462800	LOC_Os02g26430	**WRKY42**		WRKY42	WRKY42	WRKY42	WRKY50	27	WRKY42	WRKY42	WRKY50	WRKY42	WRKY42
45	Os05g0567200	LOC_Os05g49210	**WRKY43**		WRKY43	WRKY43	WRKY43	WRKY86	59	WRKY43	WRKY43	WRKY86		WRKY43
46	Os05g0322900	LOC_Os05g25770	**WRKY45**		WRKY45	WRKY45	WRKY45	WRKY71	50	WRKY45	WRKY45	WRKY71	WRKY45/WRKY71	WRKY45
47	Os11g0116900	LOC_Os11g02480	**WRKY46**	**WRKY91**	WRKY46	WRKY46	WRKY46	WRKY76	88	WRKY46	WRKY46A	WRKY76	WRKY46a	WRKY46A
48	Os07g0680400	LOC_Os07g48260	**WRKY47**		WRKY47	WRKY47	WRKY47	WRKY79	72	WRKY47	WRKY47	WRKY79	WRKY47	WRKY47
49	Os05g0478400	LOC_Os05g40060	**WRKY48**		WRKY48	WRKY48	WRKY48	WRKY95	53	WRKY48	WRKY48	WRKY95		WRKY48
50	Os05g0565900	LOC_Os05g49100	**WRKY49**		WRKY49	WRKY49	WRKY49	WRKY85	58	WRKY49	WRKY49	WRKY85	WRKY49/WRKY85	WRKY49
51	Os11g0117600	LOC_Os11g02540	**WRKY50**		WRKY50	WRKY50	WRKY50	WRKY11	91	WRKY50	WRKY50	WRKY11	WRKY50	WRKY50
52	Os04g0287400	LOC_Os04g21950	**WRKY51**		WRKY51	WRKY51	WRKY51	WRKY42	40	WRKY51	WRKY51	WRKY42	WRKY51/WRKY42	WRKY51
53	Os11g0116600	LOC_Os11g02470	**WRKY52**		WRKY52	WRKY52	WRKY52	WRKY7	87	WRKY52	WRKY52	WRKY7		WRKY52
54	Os05g0343400	LOC_Os05g27730	**WRKY53**		WRKY53	WRKY53	WRKY53	WRKY61	51	WRKY53	WRKY53	WRKY61	WRKY53/WRKY61	WRKY53
55	Os05g0478800	LOC_Os05g40080	**WRKY54**		WRKY54	WRKY54	WRKY54	WRKY93	56	WRKY54	WRKY54	WRKY93	WRKY54	WRKY54
56	Os03g0321700	LOC_Os03g20550	**WRKY55**		WRKY55	WRKY55	WRKY55	WRKY72	31	WRKY55	WRKY55	WRKY72	WRKY55	WRKY55
57	Os01g0843800	LOC_Os01g62514	**WRKY56**		WRKY56	WRKY56/92	WRKY56			WRKY56		WRKY108	WRKY101/WRKY1	WRKY101
58	Os12g0102300	LOC_Os12g01180	**WRKY57**		WRKY57	WRKY57	WRKY57	WRKY73	95	WRKY57	WRKY57	WRKY73		WRKY57
59	Os05g0528500	LOC_Os05g45230	**WRKY58**		WRKY58	WRKY58	WRKY58	WRKY108		WRKY58	WRKY58	WRKY112	WRKY58/WRKY11	WRKY58
60	Os03g0657400	LOC_Os03g45450	**WRKY60**		WRKY60	WRKY60	WRKY60	WRKY25	34	WRKY60	WRKY60	WRKY25		WRKY60
61	Os11g0685700	LOC_Os11g45850	**WRKY61**	**WRKY103**	WRKY61	WRKY61	WRKY61	WRKY66	94	WRKY61	WRKY61	WRKY66	WRKY61/WRKY78	WRKY61
62	Os09g0417800	LOC_Os09g25070	**WRKY62**		WRKY62	WRKY62	WRKY62	WRKY14	82	WRKY62	WRKY62	WRKY14	WRKY62/WRKY14	WRKY62
63		LOC_Os11g45920	**WRKY63**		WRKY63		WRKY63	WRKY67	93	WRKY63		WRKY67		

A	B	C	D	E	F	G	H	I	J	K	L	M	N	O
No.	RAP_ID	MSU(TIGR)_ID	**CGSNL proposal main gene symbol**	**CGSNL proposal synonym gene symbol**	Zhang et al. 2004 PMID: 15047897	Qiu et al. 2004 **DOI: 10.1360/982004-183**	Xie et al. 2005 PMID: 15618416	Zhang & Wang 2005 PMID: 15629062	Wu et al. 2005 PMID= 16106749	Ryu et al. 2006 PMID: 16528562	Ross et al. 2007 **DOI:10.1111/j.1744-7909.2007.00504.x**	Berri et al. 2009 PMID: 19772648	Rushton 2010 review(tableS1)	Rushton 2010 review(tableS2)
64	Os12g0116700	LOC_Os12g02450	**WRKY64**		WRKY64	WRKY64	WRKY64	WRKY75	97	WRKY64	WRKY64	WRKY75	WRKY64	WRKY64
65	Os12g0116850	LOC_Os12g02470	**WRKY65**		WRKY65	WRKY65	WRKY65	WRKY74	96	WRKY65	WRKY65	WRKY74		WRKY65
66	Os02g0698800	LOC_Os02g47060	**WRKY66**		WRKY66	WRKY66	WRKY66	WRKY57	29	WRKY66	WRKY66	WRKY57	WRKY66	WRKY66
67	Os05g0183100	LOC_Os05g09020	**WRKY67**		WRKY67	WRKY67	WRKY67	WRKY63	47	WRKY67	WRKY67	WRKY63	WRKY67/ WRKY63	WRKY67
68	Os04g0605100	LOC_Os04g51560	**WRKY68**		WRKY68	WRKY68	WRKY68	WRKY30	44	WRKY68	WRKY68	WRKY30	WRKY68	WRKY68
69	Os08g0386200	LOC_Os08g29660	**WRKY69**		WRKY69	WRKY69	WRKY69	WRKY53	75	WRKY69	WRKY69	WRKY53	WRKY69/ WRKY53	WRKY69
70	Os05g0474800	LOC_Os05g39720	**WRKY70**		WRKY70	WRKY70	WRKY70	WRKY69	52	WRKY70	WRKY70	WRKY69	WRKY70/ WRKY69	WRKY70
71	Os02g0181300	LOC_Os02g08440	**WRKY71**		WRKY71	WRKY71	WRKY71	WRKY18	25	WRKY71	WRKY71	WRKY18	WRKY71 / WRKY18	WRKY71
72	Os11g0490900	LOC_Os11g29870	**WRKY72**		WRKY72	WRKY72	WRKY72	WRKY78	92	WRKY72	WRKY72	WRKY78	WRKY72	WRKY72
73	Os06g0146250	LOC_Os06g05380	**WRKY73**		WRKY73	WRKY73	WRKY73	WRKY5	63	WRKY73	WRKY73	WRKY5		WRKY73
74	Os09g0334500	LOC_Os09g16510	**WRKY74**		WRKY74	WRKY74	WRKY74	WRKY100	80	WRKY74	WRKY74	WRKY100	WRKY74/ WRKY10	WRKY74
75	Os05g0321900	LOC_Os05g25700	**WRKY75**		WRKY75	WRKY75	WRKY75	WRKY70	49	WRKY75	WRKY75	WRKY70		WRKY75
76	Os09g0417600	LOC_Os09g25060	**WRKY76**		WRKY76	WRKY76	WRKY76	WRKY58	81	WRKY76	WRKY76	WRKY58	WRKY76	WRKY76
77	Os01g0584900	LOC_Os01g40260	**WRKY77**		WRKY77	WRKY77	WRKY77	WRKY20	7	WRKY77	WRKY77	WRKY20	WRKY77/ WRKY20	WRKY77
78			**WRKY78**			WRKY78								
79	Os03g0335200	LOC_Os03g21710	**WRKY79**			WRKY79		WRKY19	32		WRKY44	WRKY19	WRKY44/ WRKY19	WRKY44
80	Os03g0855100	LOC_Os03g63810	**WRKY80**			WRKY80		WRKY81	30		WRKY88	WRKY81	WRKY88	WRKY88
81	Os03g0444900	LOC_Os03g33012	**WRKY81**			WRKY81				WRKY84	WRKY84	WRKY107	WRKY84/ WRKY1	WRKY84
82	Os05g0233100	LOC_Os05g14370	**WRKY82**			WRKY82		WRKY45	48		WRKY90	WRKY45		WRKY90
83	Os05g0583600	LOC_Os05g50680	**WRKY83**			WRKY83								
84	Os05g0478700	LOC_Os05g40070	**WRKY84**			WRKY84		WRKY94	54		WRKY91	WRKY94		WRKY91
85			**WRKY85**			WRKY85								
86			**WRKY86**			WRKY86								
87	Os07g0583700	LOC_Os07g39480	**WRKY87**			WRKY87	WRKY78	WRKY59	70	WRKY78	WRKY78	WRKY59	WRKY78	WRKY78
88	Os07g0596900	LOC_Os07g40570	**WRKY88**			WRKY88		WRKY103	68		WRKY96	WRKY103	WRKY96	WRKY96
89	Os08g0276200	LOC_Os08g17400	**WRKY89**			WRKY89		WRKY48	74	WRKY82	WRKY82	WRKY48	WRKY82	WRKY82
90	Os09g0481700	LOC_Os09g30400	**WRKY90**			WRKY90	WRKY80	WRKY13	83	WRKY80	WRKY80	WRKY13	WRKY80	WRKY80
91	Os11g0116900	LOC_Os11g02480	**WRKY46**	**WRKY91**		WRKY91		WRKY8	88			WRKY8		
92			**WRKY92**			WRKY92								
93			**WRKY93**			WRKY93								
94	Os12g0597700	LOC_Os12g40570	**WRKY94**			WRKY94		WRKY89	102	WRKY83	WRKY83	WRKY89	WRKY83	WRKY83

A	B	C	D	E	F	G	H	I	J	K	L	M	N	O
No.	RAP_ID	MSU(TIGR)_ID	**CGSNL proposal main gene symbol**	**CGSNL proposal synonym gene symbol**	Zhang et al. 2004 PMID: 15047897	Qiu et al. 2004 **DOI: 10.1360/982004-183**	Xie et al. 2005 PMID: 15618416	Zhang & Wang 2005 PMID: 15629062	Wu et al. 2005 PMID=16106749	Ryu et al. 2006 PMID: 16528562	Ross et al. 2007 **DOI:10.1111/j.1744-7909.2007.00504.x**	Berri et al. 2009 PMID: 19772648	Rushton 2010 review(tableS1)	Rushton 2010 review(tableS2)
95	Os12g0116600	LOC_Os12g02440	**WRKY95**			WRKY95		WRKY101	98		WRKY56	WRKY101	WRKY56/WRKY10	WRKY56
96	Os12g0507300	LOC_Os12g32250	**WRKY96**			WRKY96		WRKY83	101	WRKY85	WRKY85	WRKY83	WRKY85	WRKY85
97	Os12g0116400	LOC_Os12g02420	**WRKY97**			WRKY97			99		WRKY46B		WRKY46b	WRKY46b
98			**WRKY98**			WRKY38								
99			**WRKY99**			WRKY44								
100			**WRKY100**			WRKY59								
101			**WRKY101**			WRKY63								
102	Os01g0182700	LOC_Os01g08710	**WRKY102**				WRKY79	WRKY90	1	WRKY79	WRKY79	WRKY90	WRKY79/WRKY90	WRKY79
103	Os11g0685700	LOC_Os11g45850	**WRKY61**	**WRKY103**			WRKY81		94	WRKY81				
104	Os11g0117400	LOC_Os11g02520	**WRKY104**					WRKY9	89		WRKY100	WRKY9	WRKY100/WRKY8	WRKY100
105	Os08g0198000	LOC_Os08g09800	**WRKY105**					WRKY15				WRKY15		transposon
106	Os08g0198100	LOC_Os08g09810	**WRKY106**					WRKY16				WRKY16		transposon
107	Os01g0185900	LOC_Os01g09080	**WRKY107**					WRKY26	2		WRKY97	WRKY26	WRKY97	WRKY97
108	Os01g0821300	LOC_Os01g60600	**WRKY108**					WRKY41	20		WRKY86	WRKY41		WRKY86
109	Os05g0129800	LOC_Os05g03900	**WRKY109**					WRKY52	45		WRKY89	WRKY52	WRKY89	WRKY89
110			**WRKY110**					WRKY55				WRKY55		
111	Os05g0584000	LOC_Os05g50700	**WRKY111**					WRKY60	62		WRKY92	WRKY60		WRKY92
112	Os09g0269300	LOC_Os09g09630	**WRKY112**					WRKY62	77		WRKY98	WRKY62	WRKY98	WRKY98
113	Os06g0158100	LOC_Os06g06360	**WRKY113**					WRKY64	64		WRKY93	WRKY64	WRKY93/WRKY64	WRKY93
114	Os12g0116100	LOC_Os12g02400	**WRKY114**					WRKY77	100		WRKY81	WRKY77	WRKY81	WRKY81
115	Os07g0460900	LOC_Os07g27670	**WRKY115**					WRKY87	69		WRKY95	WRKY87	WRKY95	WRKY95
116	Os01g0820700	LOC_Os01g60520	**WRKY116**					WRKY105	18		WRKY18	WRKY105		WRKY18
117	Os08g0198400	LOC_Os08g09840	**WRKY117**					WRKY109				WRKY113		transposon
118	Os08g0198900	LOC_Os08g09900	**WRKY118**					WRKY111				WRKY115		transposon
119	Os01g0842700	LOC_Os01g62510	**WRKY119**						23			WRKY119		lack WRKY signature
120			**WRKY120**						33					
121	Os03g0741400	LOC_Os03g53050	**WRKY121**						35		WRKY87	WRKY109	WRKY87	WRKY87
122	Os03g0758900	LOC_Os03g55164	**WRKY4**	**WRKY122**					36			WRKY120		
123	Os07g0273700	LOC_Os07g17230	**WRKY123**						71		WRKY94			WRKY94(lacks WRKY signature)
124			**WRKY124**						85		WRKY99			
125	Os11g0684100	LOC_Os11g45750	**WRKY125**								WRKY59			WRKY59(lack of WRKY signature)
